# Comparison of the 2021 EUGOGO guidelines and the 2022 ATA/ETA consensus statement for the management of Graves’ orbitopathy

**DOI:** 10.1530/ETJ-25-0318

**Published:** 2025-12-08

**Authors:** Luigi Bartalena, Daniela Gallo, George J Kahaly, Michele Marinò, Maria Laura Tanda

**Affiliations:** ^1^ School of Medicine, University of Insubria, Varese, Italy; ^2^ Department of Medicine and Surgery, University of Insubria, Varese, Italy; ^3^ Department of Medicine I, Johannes Gutenberg-University (JGU) Medical Center, Mainz, Germany; ^4^ Department of Clinical and Experimental Medicine, University of Pisa, Pisa, Italy

**Keywords:** Graves’ orbitopathy, thyroid eye disease, thyroid associated ophthalmopathy, guidelines, consensus statement

## Abstract

**Background:**

In 2021, the European Group on Graves’ orbitopathy (EUGOGO) published clinical practice guidelines for the management of GO, and in 2022, the American and European Thyroid Associations published the ATA/ETA consensus statement on the same topic.

**Aims:**

i) To highlight similarities and differences between the two documents, ii) to suggest possible amendments for future revisions.

**Results and conclusions:**

The two documents show a high degree of concordance as to classification, assessment, and prevention, as well as treatment of mild GO, moderate-to-severe and inactive GO, and sight-threatening GO. The major disagreement regards treatment of moderate-to-severe and active GO. The EUGOGO guidelines, written when teprotumumab was not available outside the USA, indicate intravenous glucocorticoids (IVGC) (with/without mycophenolate) as the first-line treatment, whereas the ATA/ETA consensus statement indicates teprotumumab as the first-line treatment for virtually all phenotypes of moderate-to-severe and active GO, particularly when exophthalmos is predominant. However, also in the ATA/ETA consensus statement, IVGC are the preferred treatment when the goal is GO inactivation and resolution of inflammation, and among the preferred treatments (in combination with orbital radiotherapy) when the goals are inactivation and correction of eye dysmotility. Recent developments to be considered in future revisions of the guidelines/consensus statement include the possible use of biologicals (teprotumumab and tocilizumab) for dysthyroid optic neuropathy, treatment of longstanding inactive GO with teprotumumab, reconsideration of the cautious use of radioactive iodine in patients with moderate-to-severe and active GO concomitantly treated with IVGC (with/without orbital radiotherapy), and use of statins as an adjunct therapy.

Graves’ orbitopathy (GO) (a.k.a. thyroid eye disease or thyroid-associated ophthalmopathy) is an autoimmune orbital disorder and the most common extrathyroidal manifestation/complication of Graves’ disease (1). GO affects about 30% of Graves’ patients in a clinically meaningful way (2), and in a small proportion of cases (6–7%), it may occur in patients with euthyroid/hypothyroid chronic autoimmune thyroiditis (3, 4). Management of GO, especially of severe forms, still constitutes a challenge. However, a better understanding of GO pathogenesis (5, 6, 7) allowed the development of biologicals targeting fundamental pathogenic pathways, with promising results (8, 9, 10).

In 2008, the European Group on Graves’ orbitopathy (EUGOGO) published the first consensus statement (11), followed by the first clinical practice guideline for the management of GO (12). The latter was revised and updated in 2021 (13). Subsequently, a task force of the American Thyroid Association (ATA) and the European Thyroid Association (ETA) published a consensus statement on this subject (14). Aims of the present article were to: i) highlight similarities and differences between the two most recent documents, ii) propose possible additions/amendments for future revisions based on recent additions to the literature.

## Format of the two documents

The EUGOGO document used the GRADE system (15) to make evidence-based recommendations (strong or weak) and to express the quality of evidence (very low, low, moderate, or high) (13). Because some medical therapies for GO have not been compared with placebo or with one another in randomized clinical trials (RCTs), the ATA/ETA task force selected the consensus statement format as the forum of experts, providing a more flexible instrument which recognizes the polymorphism of clinical situations and tries to tailor personalized treatments based on individual clinical features and their known responsiveness to available therapies (14). Accordingly, recommendations of the key points were indicated as ‘preferred treatment’, ‘acceptable treatment’, and ‘may be considered’ (14).

### Comment

The EUGOGO guidelines share limitations and challenges in developing strong recommendations based on low-quality evidence, as discussed by coauthors of several ATA guidelines (16) in response to critiques raised to their recommendations (17). Of the 32 EUGOGO recommendations, 27 were strong and 5 weak; while all five weak recommendations had a low level of evidence, among the 27 strong recommendations, only six had a high level of evidence, the remaining being judged as having moderate (*n* = 13) or low (*n* = 8) level of evidence (13). The ATA/ETA consensus statement task force prudently avoided rigid recommendations, taking the above limitations into consideration and following the concept that one treatment does not fit all patients (14). However, their indications, as the EUGOGO guidelines’ recommendations, could not avoid being influenced by the large clinical experience of the members of the two task forces, something that should not be discarded.

## Referral of patients with GO to specialized centers

It is widely accepted that early diagnosis and referral of patients with GO to specialized centers are fundamental because the efficacy of treatments for severe forms of GO is usually inversely related to the duration of the disease (18). A possible exception to this rule may be represented by teprotumumab, which in an RCT appeared to be effective also in GO of longer duration and with limited signs of activity (19). The EUGOGO guidelines recommend that, with the exception of the mildest cases, all Graves’ patients with overt GO (or even without GO but with risk factors for its occurrence/progression) should be referred to specialized centers (thyroid-eye clinics) for an early, timely, and multidisciplinary general treatment plan, not only for GO but also for the associated hyperthyroidism (13) (Table 1). Although the ATA/ETA consensus statement recognizes the value of the thyroid-eye clinic model or similar organizational systems, the task force limits the early and urgent referral to specialists with specific expertise in the management of GO only to cases of severe and/or sight-threatening GO (14) (Table 1).

**Table 1:** Comparison of the main recommendations of the EUGOGO guidelines and the ATA/ETA consensus statement.

Issue	EUGOGO guidelines (2021)	ATA/ETA consensus statement (2022)
Referral of patients	Early referral to thyroid-eye clinics/specialized centers, except for the mildest cases of GO	Urgent referral to specialists limited to sight-threatening (very severe) GO
Prevention	Control of risk factors	The same
Classification	Activity: clinical activity score (active, inactive) Severity: mild, moderate-to-severe, sight-threatening (very severe)	The same, but more emphasis on the VISA scoring More detailed information on formal ophthalmological examination
Management of mild GO	Watchful strategy Control of risk factors Local treatments (eye drops, ophthalmic gels) Selenium course	The same
Moderate-to-severe and active GO	Intravenous glucocorticoids (IVGC) (+/− mycophenolate) as first-line treatment Second-line treatments (including teprotumumab) if response is poor	Teprotumumab preferred (first-line) treatment in all conditions, except when inactivation/soft tissue involvement is the only goal IVGC preferred treatment when inactivation/soft tissue involvement is the goal of treatment IVGC (with/without orbital radiotherapy) as one of the preferred treatment when eye dysmotility is the target
Moderate-to-severe and inactive GO	Rehabilitative surgery	The same
Sight-threatening GO	Intravenous glucocorticoids and/or urgent decompression	The same Possible combination of IVGC and orbital radiotherapy
Treatment of hyperthyroidism	Mild and inactive GO: any treatment (ATDs, RAI, thyroidectomy) Mild and active GO: any treatment. If RAI is selected: steroid prophylaxis Moderate-to-severe and inactive GO: any treatment Moderate-to-severe and active GO: avoid RAI Sight-threatening GO: ATDs	Treatment of hyperthyroidism is only briefly addressed Steroid prophylaxis in mild and active GO Avoid RAI in moderate-to-severe and active GO

### Comment

The different approach probably reflects, at least in part, the difficulty of organizing structured thyroid-eye clinics in several countries, including the USA. Guidance for referral pathways and management outside specialized centers has been proposed in the UK (20), but satisfaction of patients seems to be greater in those who attended thyroid-eye clinics/specialized centers (21, 22). Furthermore, the management of GO is strictly connected with the treatment of Graves’ hyperthyroidism (23), reinforcing the concept that a multidisciplinary approach involving endocrinologists and ophthalmologists is warranted for Graves’ hyperthyroidism complicated by GO.

## Prevention

GO results from a complex interplay of endogenous factors (age, male gender, and still incompletely defined genetic factors) (24) and exogenous risk factors (2). The latter include cigarette smoking, poorly controlled thyroid dysfunction (both hyperthyroidism and hypothyroidism), radioactive iodine (RAI) treatment, oxidative stress, high TSH-receptor antibody (TSHR-Ab) levels, diabetes, and dyslipidemia (2). At the onset of Graves’ hyperthyroidism, it is difficult to predict whether a patient without GO will develop GO, and a predictive score proposed by EUGOGO can identify sufficiently well newly diagnosed Graves’ patients who will not manifest GO later on, rather than those who will (25).

### Comment

The EUGOGO guidelines and the ATA/ETA consensus statement agree, with no substantial differences, that active measures should be taken to control risk factors, including urging patients to quit smoking, rapid restoration and stable maintenance of euthyroidism, a course of selenium in patients with mild GO (see below), and use of low-dose prednisone (steroid prophylaxis) in patients with mild GO (or risk factors for GO occurrence) receiving RAI treatment (13, 14).

## Assessment and classification

GO is considered a self-limiting disease, with an initial inflammatory phase (active GO), followed by stabilization and eventually by incomplete regression (inactive GO) (18). The degree of inflammation caused by autoimmune reactions ongoing in the orbit is responsible for the overall severity of ocular manifestations. Thus, *activity* and *severity* are the two features to be considered in the assessment. Classification of GO into mild, moderate-to-severe, and sight-threatening (very severe), either active or inactive, initially proposed by EUGOGO in 2006 (26), is accepted by both documents (13, 14). The activity of GO is widely evaluated by the clinical activity score (CAS) (27), although, as underscored by the ATA/ETA consensus statement, CAS has several limitations, including its binary (yes/no) classification, assignment of the same weight (one point) to manifestations of different severity/impact, inclusion of subjective features, and possible false-positive (congestive rather than inflammatory signs) and false-negative (‘white eye’ phenotype) information (14). Assessment of severity requires a thorough ophthalmological evaluation, including exophthalmometer reading, measurement of lid aperture, assessment of visual acuity and ocular motility, and corneal evaluation (13, 14). Changes in the severity of GO with time help identify patients with persistently active disease. An alternative scoring system, less validated than the EUGOGO classification, is the VISA (Vision, Inflammation, Strabismus, Appearance) (28). Although, as indicated by the EUGOGO guidelines, imaging by MRI may be of some help in predicting the subsequent response to immunosuppressive treatments for moderate-to-severe and active GO (13), imaging (either MRI or non-contrast CT scan) should be limited to assist diagnosis in doubtful cases (e.g., unilateral or asymmetrical cases), to identify patients at risk of dysthyroid optic neuropathy (DON) by showing apical crowding, and before orbital decompression and squint surgery (14). Because GO heavily affects quality of life (QoL), the initial assessment of a patient with GO should always consider evaluation of this important feature. According to both documents, the instrument which has received the widest validation (and translation into several languages) is the GO-QoL (29).

### Comment

There is no substantial difference between the two documents regarding classification and assessment (Table 1). The ATA/ETA consensus statement emphasizes, more than the EUGOGO guidelines, the role of the VISA instrument for a quick assessment of both activity and severity of GO (14). At variance with the EUGOGO guidelines, the ATA/ETA consensus statement provides detailed information on the ophthalmological examination techniques (14). The complexity of the assessment further underscores the need for early referral of Graves’ patients with GO to thyroid-eye clinics or specialized centers with expertise in this field for an optimal treatment plan.

## Management of mild GO

When GO is mild, the two documents agree on a wait-and-see strategy, the use of topical agents (artificial tears during the day, ocular gels at nighttime), a 6-month course of selenium, particularly in selenium-deficient areas (30), as well as on general measures to prevent progression of GO or favor its regression (e.g. smoking cessation, control of thyroid dysfunction) (13, 14). The Mediterranean diet, naturally enriched in selenium as well as in many antioxidants with anti-inflammatory properties, might have some beneficial effect on GO (31). In selected cases with a significant impact on QoL, a course of low-dose oral glucocorticoids (OGC), or in inactive forms, rehabilitative surgery can be proposed according to both documents (13, 14).

### Comment

There is no difference between the two documents regarding the management of mild GO (Table 1). Both state that evidence is limited regarding the use of selenium in selenium-sufficient areas, and a recent RCT from selenium-sufficient Korea showed a marginal benefit of selenium supplementation at 3 months, but not at 6-month follow-up (32). In addition, both documents underscore the lack of evidence for the use of selenium in moderate-to-severe forms of GO, although this inappropriate approach is not infrequent in clinical practice (33).

## Management of moderate-to-severe GO

### Moderate-to-severe and active GO

This is the section of the documents showing the highest degree of disagreement (Table 1). The EUGOGO guidelines indicate intravenous glucocorticoids (IVGC), with or without mycophenolate, as the first-line treatment for moderate-to-severe and active GO, and a number of options (a second course of IVGC, OGC with cyclosporine or azathioprine, orbital radiotherapy with OGC or IVGC, biologicals, including teprotumumab, rituximab, and tocilizumab) as second-line treatments in case of poor response to or deterioration after the first-line treatment (13). High-dose GC have been used for these forms of GO for more than 60 years, and the use of mycophenolate was supported, at the time the EUGOGO guidelines were written, by two RCTs (34, 35). Although the Chinese paper (34) was subsequently retracted, other studies, including one RCT, have shown beneficial effects of mycophenolate alone or in combination with GC (36, 37).

In the ATA/ETA consensus statement, IVGC are i) the preferred treatment when the goals of treatment are disease inactivation and resolution of inflammatory soft tissue changes, and ii) one of the preferred treatments (multiple options), alone or in combination with orbital radiotherapy, when the treatment goal is improvement in ocular motility and reduction of inflammation (14). In the ATA/ETA consensus statement, IVGC are i) the preferred treatment when the goals of treatment are disease inactivation and resolution of inflammatory soft tissue changes, and ii) one of the preferred treatments (multiple options), alone or in combination with orbital radiotherapy, when the treatment goal is improvement in ocular motility (14). Based on the results of two RCTs (38, 39) and one paper evaluating the pooled data from the two RCTs (40), teprotumumab is the preferred (first-line) treatment when treatment goals are i) disease inactivation and diplopia, or ii) reduction of exophthalmos; and one of the preferred treatments (multiple options) when eye motility improvement is the goal (14). According to the ATA/ETA consensus statement, rituximab and tocilizumab may be considered when the treatment goal is disease inactivation and soft tissue changes, but not as the first-line treatment (14).

### Comment

At the time the EUGOGO guidelines were published, teprotumumab was not available outside the USA, where it was approved by the FDA in January 2020. Following recommendation by the European Medicines Agency (EMA), marketing authorization in the European Union was issued in June 2025. The lack of drug availability and accessibility, as well as its exceedingly high cost and the lack of solid data on teprotumumab durability and safety, convinced the EUGOGO task force to include teprotumumab among second-line drugs. Now more data have accumulated, indicating that teprotumumab is effective, particularly on exophthalmos, but also on diplopia, CAS, and QoL (41, 42, 43). However, about 30% of patients do not maintain the response in terms of exophthalmos, diplopia, and overall response 51 weeks after treatment completion (41), suggesting that teprotumumab is, as all the other currently available drugs for GO, a disease-modulating rather than a disease-modifying agent (44). In addition, concerns remain regarding adverse events of teprotumumab, in particular hyperglycemia and hearing impairment (45, 46, 47, 48, 49, 50). Looking at the ATA/ETA consensus statement, it seems that IVGC (alone or in combination with orbital radiotherapy) can still be proposed as the preferred treatment (as an alternative to teprotumumab) when inflammatory signs/symptoms are predominant, but also when eye dysmotility, generally causing diplopia, is present (14). Admittedly, the effect of IVGC on exophthalmos is modest (51). It should be mentioned that RCTs directly comparing teprotumumab with IVGC (with/without orbital radiotherapy) are still missing.

### Moderate-to-severe and inactive GO

Very little space is dedicated by the EUGOGO guidelines to the management of GO in this phase of the disease. In particular, surgical rehabilitative therapies to correct residual exophthalmos, eye muscle dysfunction, and eyelid malposition are indicated, without going into details of surgical approaches (13). The document indicated that an *ad hoc* document was going to be prepared by the ophthalmologist members of EUGOGO, but this has not been published yet. The ATA/ETA consensus statement contains the same indications for surgeries, but provides a more detailed presentation of surgical techniques (14).

### Comment

Rehabilitative surgery still represents an important step in the overall management of GO and, with the exception of emergency decompression, is elective and confined to the inactive forms of GO, with a staged approach, shared by the two documents, which includes orbital decompression first, followed by eye muscle surgery, and eventually by eyelid surgery, as needed.

## Management of sight-threatening (very severe) GO

Sight-threatening forms of GO are rare and may be due to either DON or corneal breakdown. Both represent an emergency and should be treated without delay. Both documents agree on the use of high-dose IVGC as the first-line approach for DON, but orbital decompression should be promptly performed if response is poor/absent or deterioration occurs (13, 14) (Table 1). However, the ATA/ETA indicates that the combination of IVGC with orbital radiotherapy is an acceptable treatment and a possibly surgery-sparing alternative (14).

### Comment

Whether orbital radiotherapy is really useful in this clinical setting is controversial and is so far based only on retrospective studies (52, 53). In addition, exacerbation of DON by orbital radiotherapy has been reported (54).

## Treatment of hyperthyroidism in patients with GO

The EUGOGO guidelines devoted an entire paragraph and five recommendations (#28–32) to the treatment of hyperthyroidism in patients with GO (13). The latter can be summarized as follows (Table 1): i) in patients with no GO or mild and inactive GO, any treatment for hyperthyroidism (antithyroid drugs (ATDs), RAI, or thyroidectomy) can be chosen on the basis of standardized criteria and patient choice; ii) in patients with mild and active GO, if RAI treatment is selected, steroid prophylaxis with low-dose prednisone should be applied to prevent progression of GO; iii) in patients with moderate-to-severe, longstanding, and inactive GO, if RAI treatment is used, steroid prophylaxis should be considered in patients at risk of disease flare (e.g., smokers); iv) in patients with moderate-to-severe and active GO, ATDs are the preferred treatment and RAI treatment should be avoided; v) sight-threatening GO due to DON is an emergency requiring immediate medical and/or surgical treatment, during which hyperthyroidism should be controlled by ATDs (13). The ATA/ETA consensus statement does not specifically discuss the problem of treatment of hyperthyroidism in patients with GO, except for briefly mentioning that euthyroidism should be promptly restored and stably maintained, RAI treatment should be avoided in patients with moderate-to-severe and active GO, and should be associated, under most circumstances, with steroid prophylaxis in patients with mild GO (14).

### Comment

Treatment of Graves’ hyperthyroidism in patients with moderate-to-severe and active GO is a controversial issue (10, 23). A recent international survey among thyroid specialists showed that ATDs are, worldwide and by far, the preferred treatment (68% of respondents) in this clinical setting, followed by surgery (23%), and, finally, by RAI (9%, with or without steroid prophylaxis) (55). Reluctance to use RAI treatment in moderate-to-severe and active GO derives from RCTs showing that RAI treatment can cause *de novo* occurrence or progression of mild GO (56, 57, 58), although this untoward effect can almost always be prevented by low-dose prednisone prophylaxis (57, 59).

## Considerations for revision/amendments of current recommendations

After decades of slow and limited progress, management of GO is now a rapidly evolving field, many agents are under active investigation (9), there is a sort of race for treatment (60), and guidelines/consensus statements may need to be updated 3–4 years after their publication, but run the risk of becoming quickly outdated, at least in part.

A revised document, either guidelines or a consensus statement, should take into consideration recent additions to the literature involving several aspects of the management of GO (Table 2).

**Table 2:** Suggestions for amendments/revisions of current guidelines for the management of GO.

Issue	Suggestions
Dyslipidemia	Correct dyslipidemia with statins Possible preventive use of statins independently of lipid profile Possible use of statins in association with intravenous glucocorticoids (IVGC) for moderate-to-severe and active GO
Inactive GO	Teprotumumab as an acceptable treatment
Dysthyroid optic neuropathy (DON)	Teprotumumab and tocilizumab as acceptable treatments (especially in IVGC- and/or surgery-resistant DON)
Treatment of Graves’ hyperthyroidism	Use of radioactive iodine treatment in moderate-to-severe and active GO if the latter is concomitantly treated with IVGC with/without orbital radiotherapy

### Statins

Several studies and reviews have outlined the association of dyslipidemia with GO (6, 10). Cross-sectional and retrospective studies showed a correlation between high cholesterol levels and the occurrence of GO, and a correlation between CAS, total cholesterol, and low-density lipoprotein (LDL)-cholesterol (61, 62). These results were confirmed by a large retrospective study from Brazil (63). High LDL-cholesterol levels were associated with a less favorable response of GO to IVGC (64). Two large retrospective studies found that the incidence of GO was lower in statin users than in statin non-users (65, 66). A recent nationwide, population-based retrospective study from Taiwan reported that initiating statin treatment within 1 year of the diagnosis of GD was associated with a decreased risk of GO occurrence, particularly moderate-to-severe forms, compared with a later institution (2–3 years) of statin treatment (67). Such a preventive effect was confirmed by a large (>3,500 patients) retrospective case–control study showing an 80% reduced risk of developing GO in statin users, independently of the lipid profile (68). In a phase II RCT of 88 patients with moderate-to-severe and active GO, the addition of 20 mg atorvastatin to IVGC was associated with a better overall response at 12 and 24 weeks, and a lower relapse rate after treatment withdrawal (69). Although further prospective studies or RCTs are needed, available evidence to be considered in future recommendations indicates that i) dyslipidemia is an established risk factor for GO; ii) occurrence of GO, especially moderate-to-severe forms, might be preventable by early statin treatment; iii) statins may represent a useful adjunct to the treatment of moderate-to-severe and active GO with IVGC (Table 2).

### Inactive GO

Residual manifestations of GO when the disease has been inactivated by medical treatments have traditionally been treated surgically, as needed, because of the poor effectiveness of GC and other immunosuppressive treatments in this clinical setting (1). An observational study of 31 GO patients with a mean duration of disease >2 years and CAS <3 who were treated with teprotumumab reported a decrease in exophthalmos of >3 mm and an improvement in diplopia in 67% of cases (70). Subsequently, a placebo-controlled RCT of 62 patients (CAS ≤1, mean duration of disease 5 years) showed a reduction of exophthalmos of 2.4 mm in patients treated with teprotumumab vs 0.9 mm with placebo, although the effect on diplopia was not significant (19). It is evident that these interesting findings need to be confirmed, but consideration might be given in future revisions of the guidelines/consensus statement to the use of teprotumumab in long-standing, minimally active GO with residual exophthalmos (Table 2).

### DON

The two documents indicate IVGC and/or orbital decompression as the therapies that should be urgently used for DON (13, 14). The ATA/ETA consensus statement also considers orbital radiotherapy in combination with IVGC as an acceptable treatment, despite the absence of RCTs demonstrating the effectiveness of orbital radiotherapy in this clinical setting (14). Neither document suggests the possible use of biologicals for DON. After publication of the two documents, small series/case reports have been published showing the effectiveness of either teprotumumab (71, 72, 73) or the anti-interleukin-6 receptor tocilizumab (74) on DON, even when resistant to IVGC and/or orbital decompression. Therefore, although larger and controlled studies are warranted, teprotumumab and tocilizumab might be considered for DON, especially if the latter is resistant to steroids and/or surgery (Table 2).

### Treatment of hyperthyroidism

Treatment of hyperthyroidism in patients with GO is a matter of debate, especially when GO is moderate-to-severe and active (23). While in the absence of GO or when GO is mild or moderate-to-severe but inactive, all available therapies for hyperthyroidism (ATDs, RAI, thyroidectomy) can be used, both the EUGOGO guidelines and the ATA/ETA consensus statement recommend that RAI should be avoided in the presence of moderate-to-severe and active GO (13, 14). However, a post hoc analysis of three RCTs may cast doubts on this dogma. In a single-masked RCT, 82 Graves’ patients were treated with RAI for hyperthyroidism 1 week before initiating treatment with either oral GC (*n* = 41) or IVGC (*n* = 41) combined with orbital radiotherapy for moderate-to-severe and active GO (75). Progression of GO at the last follow-up visit was observed in two patients in the oral GC group, but in no patient in the IVGC group (75). In a second RCT of 60 patients with moderate-to-severe and active GO treated with IVGC as monotherapy, hyperthyroidism was managed by total thyroidectomy alone (*n* = 30) or followed by RAI (*n* = 30) 2 weeks before initiating treatment for GO (76). Three patients in the total thyroidectomy-alone group (10%) and two patients in the total thyroidectomy-and-RAI group (6.7%) had a progression of GO at the 3-month evaluation; however, at 9 months, no patient in the total thyroidectomy-and-RAI group had a progression of GO compared with baseline (76). Finally, in a third RCT of 40 patients with moderate-to-severe and active GO, hyperthyroidism was treated by thyroidectomy alone (*n* = 20) or combined with RAI treatment (*n* = 20) before IVGC treatment for GO (77). No progression of GO was found at 3, 6, and 12 months in the group treated by thyroidectomy and RAI, while in the group treated by thyroidectomy alone, progression occurred in two patients at 6 months and three patients at 12 months (77). A recent large retrospective study supports the results of the above RCTs (78). Therefore, in future revisions of the guidelines for the management of GO, the use of RAI might be considered also in patients with moderate-to-severe and active GO, provided that treatment for GO is concomitantly instituted (Table 2). Needless to say, RAI treatment should be avoided in sight-threatening GO, because emergency management of GO and control of hyperthyroidism with ATDs are the absolute priorities.

## Conclusions

The EUGOGO guidelines and the ATA/ETA consensus show a large degree of concordance on most aspects of the management of GO. Discordance exists especially on the approach to patients with moderate-to-severe and active GO (IVGC vs teprotumumab as the first-line treatment), particularly when exophthalmos is the predominant manifestation. This discordance might be at least partially overcome by the recent approval by the EMA of teprotumumab for the management of GO. Availability is not synonymous with accessibility, as the very high cost of the drug can be covered by insurance-based health systems, but is not sustainable for many European national health systems. In addition, IVGC have been used for decades, they are effective on most features of GO except for their modest effectiveness on exophthalmos, and we learned how to select patients and to prevent and/or manage IVGC adverse events. An important and unsolved issue is represented by adverse events of teprotumumab, particularly diabetes and ototoxicity. All these aspects, including cost/effectiveness evaluation, should be considered in future revisions of guidelines/consensus statements. After the publication of the two guidelines, new evidence has been added, as delineated in this paper, which deserves adequate consideration. It is conceivable that in the near future other biologicals or agents (e.g. small molecules targeting the TSHR), currently under active investigation, may be proven to have a relevant role in the management of GO, but, for the time being, solid evidence in this regard is still missing.

## Declaration of interest

The authors declare that there is no conflict of interest that could be perceived as prejudicing the impartiality of the work reported.

## Funding

This research did not receive grants from any public agency in the public, private, or not-for-profit sector.

## Author contribution statement

All the authors contributed to conceptualization, data curation, writing the original draft, and writing and editing review.

## References

[1] Bartalena L & Tanda ML. Current concepts regarding Graves’ orbitopathy. J Intern Med 2022 292 692–716. (10.1111/joim.13524) 35604323 PMC9796560

[2] Bartalena L, Gallo D, Tanda ML, et al. Thyroid eye disease: epidemiology, natural history, and risk factors. Ophthalmic Plast Reconstr Surg 2023 39 S2–S8. (10.1097/iop.0000000000002467) 38054980

[3] Leo M, Menconi F, Rocchi R, et al. Role of the underlying thyroid disease on the phenotype of Graves’ orbitopathy in a tertiary referral center. Thyroid 2015 25 347–351. (10.1089/thy.2014.0475) 25584927

[4] Kahaly GJ, Diana T, Glang J, et al. Thyroid stimulating antibodies are highly prevalent in Hashimoto’s thyroiditis and associated orbitopathy. J Clin Endocrinol Metab 2016 101 1998–2004. (10.1210/jc.2016-1220) 26964732

[5] Neag EJ & Smith TJ. 2021 update on thyroid-associated ophthalmopathy. J Endocrinol Investig 2022 45 235–259. (10.1007/s40618-021-01663-9) 34417736 PMC9455782

[6] Lanzolla G, Marinò M & Menconi F. Graves disease: latest understanding of pathogenesis and treatment options. Nat Rev Endocrinol 2024 20 647–660. (10.1038/s41574-024-01016-5) 39039206

[7] Lee ACH & Kahaly GJ. Unravelling the pathogenic mechanisms in Graves’ orbitopathy. Eur Thyroid J 2025 14 e250200. (10.1530/etj-25-0200) 40905523 PMC12474802

[8] Toro-Tobon D & Stan MN. The evolving landscape of thyroid eye disease: present and future. Eur J Endocrinol 2025 193 R15–R24. (10.1093/ejendo/lvaf156) 40794607

[9] Lee ACH & Kahaly GJ. Targeted immunotherapies for Graves’ thyroidal & orbital diseases. Front Immunol 2025 16 1571427. (10.3389/fimmu.2025.1571427) 40145088 PMC11936961

[10] Wiersinga WM, Eckstein AK & Žarković M. Thyroid eye disease (Graves’ orbitopathy): clinical presentation, epidemiology, pathogenesis, and management. Lancet Diabetes Endocrinol 2025 13 600–614. (10.1016/s2213-8587(25)00066-x) 40324443

[11] Bartalena L, Baldeschi L, Dickinson AJ, et al. Consensus statement of the European Group on Graves’ orbitopathy (EUGOGO) on management of GO. Eur J Endocrinol 2008 158 273–285. (10.1530/eje-07-0666) 18299459

[12] Bartalena L, Baldeschi L, Boboridis K, et al. The 2016 European Thyroid Association/European Group on Graves’ orbitopathy guidelines for the management of Graves’ orbitopathy. Eur Thyroid J 2016 5 9–26. (10.1159/000443828) 27099835 PMC4836120

[13] Bartalena L, Kahaly GJ, Baldeschi L, et al. The 2021 European Group on Graves’ orbitopathy (EUGOGO) clinical practice guidelines for the medical management of Graves’ orbitopathy. Eur J Endocrinol 2021 185 G43–G67. (10.1530/eje-21-0479) 34297684

[14] Burch HB, Perros P, Bednarczuk T, et al. Management of thyroid eye disease: a consensus statement by the American Thyroid Association and the European Thyroid Association. Eur Thyroid J 2022 11 e220189. (10.1530/etj-22-0189) 36479875 PMC9727317

[15] Swiglo BA, Murad MH, Schünemann HJ, et al. A case for clarity, consistency, and helpfulness: state-of-the-art clinical practice guidelines in endocrinology using the grading of recommendations, assessment, development, and evaluation system. J Clin Endocrinol Metab 2008 93 666–673. (10.1210/jc.2007-1907) 18171699

[16] Sawka AM, Alexander EK, Bianco AC, et al. Challenges in developing recommendations based on low-quality evidence in thyroid guidelines. Thyroid 2021 31 3–7. (10.1089/thy.2020.0448) 32900277

[17] Bautista-Orduno KG, Dorsey-Trevino EG, Gonzalez-Gonzalez JG, et al. American thyroid association guidelines are inconsistent with grading of recommendations assessment, development, and evaluations-A meta-epidemiologic study. J Clin Epidemiol 2020 123 180–188. (10.1016/j.jclinepi.2020.02.010) 32145366

[18] Bartalena L, Pinchera A & Marcocci C. Management of Graves’ ophthalmopathy: reality and perspectives. Endocr Rev 2000 21 168–199. (10.1210/edrv.21.2.0393) 10782363

[19] Douglas RS, Couch S, Wester ST, et al. Efficacy and safety of teprotumumab in patients with thyroid eye disease of long duration and low disease activity. J Clin Endocrinol Metab 2023 109 25–35. (10.1210/clinem/dgad637) 37925673 PMC10735297

[20] Perros P, Dayan CM, Dickinson AJ, et al. Management of patients with Graves’ orbitopathy: initial assessment, management outside specialised centres and referral pathways. Clin Med 2015 15 173–178. (10.7861/clinmedicine.15-2-173) PMC4953738 25824071

[21] Estcourt S, Hickey J, Perros P, et al. The patient experience of services for thyroid eye disease in the United Kingdom: results of a nationwide survey. Eur J Endocrinol 2009 161 483–487. (10.1530/eje-09-0383) 19542244

[22] Tramunt B, Imbert P, Grunenwald S, et al. Sight-threatening Graves’ orbitopathy: twenty years’ experience of a multidisciplinary thyroid-eye outpatient clinic. Clin Endocrinol 2019 90 208–213. (10.1111/cen.13880) 30339291

[23] Bartalena L & Smith TJ. Treatment of hyperthyroidism in Graves’ disease complicated by thyroid eye disease. J Clin Endocrinol Metab 2025 110 922–930. (10.1210/clinem/dgaf009) 39787151

[24] Marinò M, Rotondo Dottore G, Menconi F, et al. Role of genetics and epigenetics in Graves’orbitopathy. Eur Thyroid J 2024 13 e240179. (10.1530/etj-24-0179) 39378053 PMC11623286

[25] Wiersinga W, Žarković M, Bartalena L, et al. Predictive score for the development or progression of Graves’ orbitopathy in patients with newly diagnosed Graves’ hyperthyroidism. Eur J Endocrinol 2018 178 635–643. (10.1530/eje-18-0039) 29650691

[26] Wiersinga WM, Perros P, Kahaly GJ, et al. Clinical assessment of patients with Graves’ orbitopathy: the European Group on Graves’ orbitopathy recommendations to generalists, specialists and clinical researchers. Eur J Endocrinol 2006 155 387–389. (10.1530/eje.1.02230) 16914591

[27] Mourits MP, Koornneef L, Wiersinga WM, et al. Clinical criteria for the assessment of disease activity in Graves’ ophthalmopathy: a novel approach. Br J Ophthalmol 1989 73 639–644. (10.1136/bjo.73.8.639) 2765444 PMC1041835

[28] Dolman PJ & Rootman J. VISA classification for Graves orbitopathy. Ophthalmic Plast Reconstr Surg 2006 22 319–324. (10.1097/01.iop.0000235499.34867.85) 16985411

[29] Terwee CB, Gerding MN, Dekker FW, et al. Development of a disease specific quality of life questionnaire for patients with Graves’ ophthalmopathy: the GO-QOL. Br J Ophthalmol 1998 82 773–779. (10.1136/bjo.82.7.773) 9924370 PMC1722683

[30] Marcocci C, Kahaly GJ, Krassas GE, et al. Selenium and the course of mild Graves’ orbitopathy. N Engl J Med 2011 364 1920–1931. (10.1056/nejmoa1012985) 21591944

[31] Le Moli R, Piticchio T, Pallotti F, et al. Mediterranean diet, selenium and Graves’ ophthalmopathy: a prospective, randomized, single-center study. Endocrine 2025 90 709–718 online ahead of print. (10.1007/s12020-025-04360-2) 40707809

[32] Ahn HY, Lee MJ, Jung KY, et al. Selenium vs control for Graves ophthalmopathy in a selenium-sufficient area: a randomized clinical trial. JAMA Ophthalmol 2025 143 287–294. (10.1001/jamaophthalmol.2024.6337) 40014353 PMC11869097

[33] Negro R, Hegedüs L, Attanasio R, et al. A 2018 European Thyroid Association survey on the use of selenium supplementation in Graves’ hyperthyroidism and Graves’ orbitopathy. Eur Thyroid J 2019 8 7–15. (10.1159/000494837) 30800636 PMC6381891

[34] Ye X, Bo X, Hu X, et al. Efficacy and safety of mycophenolate mofetil in patients with active moderate-to-severe Graves’ orbitopathy. Clin Endocrinol 2017 86 247–255 Retracted in May 2023. (10.1111/cen.13170) 27484048

[35] Kahaly GJ, Riedl M, König J, et al. Mycophenolate plus methylprednisolone versus methylprednisolone alone in active, moderate-to-severe Graves’ orbitopathy (MINGO): a randomised, observer-masked, multicentre trial. Lancet Diabetes Endocrinol 2018 6 287–298. (10.1016/s2213-8587(18)30020-2) 29396246

[36] Rajabi MT, Rafizadeh SM, Mohammadi A, et al. Mycophenolate Mofetil (CellCept®) in combination with low dose prednisolone in moderate to severe graves’ orbitopathy. Front Med 2022 9 788228. (10.3389/fmed.2022.788228) PMC8873183 35223896

[37] Li LF, Xue JL, Guan L, et al. Therapeutic outcomes of mycophenolate mofetil and glucocorticoid in thyroid-associated ophthalmopathy patients. Front Endocrinol 2023 14 1140196. (10.3389/fendo.2023.1140196) PMC10070944 37025403

[38] Smith TJ, Kahaly GJ, Ezra DG, et al. Teprotumumab for thyroid-associated ophthalmopathy. N Engl J Med 2017 376 1748–1761. (10.1056/nejmoa1614949) 28467880 PMC5718164

[39] Douglas RS, Kahaly GJ, Patel A, et al. Teprotumumab for the treatment of active thyroid eye disease. N Engl J Med 2020 382 341–352. (10.1056/nejmoa1910434) 31971679

[40] Kahaly GJ, Douglas RS, Holt RJ, et al. Teprotumumab for patients with active thyroid eye disease: a pooled data analysis, subgroup analyses, and off-treatment follow-up results from two randomised, double-masked, placebo-controlled, multicentre trials. Lancet Diabetes Endocrinol 2021 9 360–372. (10.1016/s2213-8587(21)00056-5) 33865501

[41] Kahaly GJ, Subramanian PS, Conrad E, et al. Long-term efficacy of teprotumumab in thyroid eye disease: follow-up outcomes in three cinical trials. Thyroid 2024 34 880–889. (10.1089/thy.2023.0656) 38824618

[42] Kahaly GJ, Xi A, Barretto N, et al. Teprotumumab improves quality of life in thyroid eye disease: meta-analysis and matching-adjusted indirect comparison. J Endocr Soc 2025 9 bvaf063. (10.1210/jendso/bvaf063) 40303547 PMC12037360

[43] Hiromatsu Y, Ishikawa E, Kozaki A, et al. A randomised, double-masked, placebo-controlled trial evaluating the efficacy and safety of teprotumumab for active thyroid eye disease in Japanese patients. Lancet Regionale Health West Pac 2025 55 101464. (10.1016/j.lanwpc.2025.101464) PMC11787687 39896230

[44] North VS, Dolman PJ, Garrity JA, et al. Disease modulation versus modification: a call for revised outcome metrics in the treatment of thyroid eye disease. Ophthalmic Plast Reconstr Surg 2024 40 156–160. (10.1097/iop.0000000000002591) 38285956

[45] Bartalena L, Marinò M, Marcocci C, et al. Teprotumumab for Graves’ orbitopathy and ototoxicity: moving problems from eyes to ears? J Endocrinol Investig 2022 45 1455–1457. (10.1007/s40618-022-01791-w) 35403994

[46] Stan MN & Krieger CC. The adverse effects profile of teprotumumab. J Clin Endocrinol Metab 2023 108 e654–e662. (10.1210/clinem/dgad213) 37071658 PMC10686693

[47] Shah SA, Amarikwa L, Sears CM, et al. Teprotumumab-related adverse events in thyroid eye disease: a multicenter study. Ophthalmology 2024 131 458–467. (10.1016/j.ophtha.2023.10.018) 37852417 PMC10960718

[48] Huang J, Su A, Yang J, et al. Postmarketing safety concerns of teprotumumab: a real-world pharmacovigilance assessment. J Clin Endocrinol Metab 2024 110 159–165. (10.1210/clinem/dgae417) 38878281 PMC11651674

[49] Hori K, Wenger T, Davuluru S, et al. Otologic symptoms in patients treated with teprotumumab: a multi-institutional national cohort study. Laryngoscope 2025 In press. (10.1002/lary.70045) 40814274

[50] Epperson MV, Hughes S, Valenzuela CV, et al. Otologic symptoms following teprotumumab administration in patients with thyroid eye disease. Otol Neurotol 2025 46 330–335. (10.1097/mao.0000000000004418) 39951668

[51] Bartalena L, Krassas GE, Wiersinga W, et al. Efficacy and safety of three different cumulative doses of intravenous methylprednisolone for moderate to severe and active Graves’ orbitopathy. J Clin Endocrinol Metab 2012 97 4454–4463. (10.1210/jc.2012-2389) 23038682

[52] Kazim M, Trokel S & Moore S. Treatment of acute Graves’ orbitopathy. Ophthalmology 1991 98 1443–1448. (10.1016/s0161-6420(91)32114-6) 1945322

[53] Gold KG, Scofield S, Isaacson SR, et al. Orbital radiotherapy combined with corticosteroid treatment for thyroid eye disease-compressive optic neuropathy. Ophthalmic Plast Reconstr Surg 2018 34 172–177. (10.1097/iop.0000000000001003) 29517594

[54] Hersh D & Kinnar M. Acute dysthyroid optic neuropathy exacerbated by orbital radiotherapy. Orbit 2014 33 385–387. (10.3109/01676830.2014.904378) 24841498

[55] Villagelin D, Cooper DS & Burch HB. A 2023 international survey of clinical practice patterns in the management of Graves disease: a decade of change. J Clin Endocrinol Metab 2024 109 2956–2966. (10.1210/clinem/dgae222) 38577717 PMC12102715

[56] Bartalena L, Marcocci C, Bogazzi F, et al. Use of corticosteroids to prevent progression of Graves’ ophthalmopathy after radioiodine therapy for hyperthyroidism. N Engl J Med 1989 321 1349–1352. (10.1056/nejm198911163212001) 2811943

[57] Bartalena L, Marcocci C, Bogazzi F, et al. Relation between therapy for hyperthyroidism and the course of Graves’ ophthalmopathy. N Engl J Med 1998 338 73–78. (10.1056/nejm199801083380201) 9420337

[58] Träisk F, Tallstedt L, Abraham-Nordling M, et al. Thyroid-associated ophthalmopathy after treatment for Graves’ hyperthyroidism with antithyroid drugs or iodine-131. J Clin Endocrinol Metab 2009 94 3700–3707. (10.1210/jc.2009-0747) 19723755

[59] Lai A, Sassi L, Compri E, et al. Lower dose prednisone prevents radioiodine-associated exacerbation of initially mild or absent graves’ orbitopathy: a retrospective cohort study. J Clin Endocrinol Metab 2010 95 1333–1337. (10.1210/jc.2009-2130) 20061414

[60] Dayan CM. The race for new treatments for Graves orbitopathy (thyroid eye disease). J Clin Endocrinol Metab 2024 109 e1938–e1939. (10.1210/clinem/dgad693) 38451776

[61] Sabini E, Mazzi B, Profilo MA, et al. High serum cholesterol is a novel risk factor for Graves’ orbitopathy: results of a cross-sectional study. Thyroid 2018 28 386–394. (10.1089/thy.2017.0430) 29336220

[62] Lanzolla G, Sabini E, Profilo MA, et al. Relationship between serum cholesterol and Graves’ orbitopathy (GO): a confirmatory study. J Endocrinological Invest 2018 41 1417–1423. (10.1007/s40618-018-0915-z) 29923059

[63] Cardo C, Bernardo Santos R, Pinotti Pedro Miklos AB, et al. The relationship between cholesterol levels and thyroid eye disease. Eur Thyroid J 2025 14 e240133. (10.1530/etj-24-0133) 39819487 PMC11825156

[64] Naselli A, Moretti D, Regalbuto C, et al. Evidence that baseline levels of low-density lipoproteins cholesterol affect the clinical response of Graves’ ophthalmopathy to parenteral corticosteroids. Front Endocrinol 2020 11 609895. (10.3389/fendo.2020.609895) PMC7784376 33414766

[65] Stein JD, Childers D, Gupta S, et al. Risk factors for developing thyroid-associated ophthalmopathy among individuals with Graves disease. JAMA Ophthalmol 2015 133 290–296. (10.1001/jamaophthalmol.2014.5103) 25502604 PMC4495733

[66] Nilsson A, Tsoumani K & Planck T. Statins decrease the risk of orbitopathy in newly diagnosed patients with Graves disease. J Clin Endocrinol Metab 2021 106 1325–1332. (10.1210/clinem/dgab070) 33560351

[67] Chou YT, Lai CC, Li CY, et al. Early statin use following diagnosis of Graves’ disease is associated with a reduced risk of moderate-to-severe Graves’ orbitopathy in middle-aged adults: evidence from a nationwide Taiwanese cohort. Thyroid 2025 35 1052–1062. (10.1177/10507256251364782) 40803364

[68] Hsu GC, Shih SR, Chang FY, et al. An appraisal of the preventive effect of statins on the development of Graves’ ophthalmopathy: a hospital-based cohort study. Ophthalmol Ther 2024 13 1499–1511. (10.1007/s40123-024-00930-1) 38581604 PMC11109055

[69] Lanzolla G, Sabini E, Leo M, et al. Statins for Graves’ orbitopathy (STAGO): a phase 2, open-label, adaptive, single centre, randomised clinical trial. Lancet Diabetes Endocrinol 2021 9 733–742. (10.1016/s2213-8587(21)00238-2) 34592164

[70] Ugradar S, Kang J, Kossler AL, et al. Teprotumumab for the treatment of chronic thyroid eye disease. Eye 2022 36 1553–1559. (10.1038/s41433-021-01593-z) 34244669 PMC9307784

[71] Sears CM, Wang Y, Bailey LA, et al. Early efficacy of teprotumumab for the treatment of dysthyroid optic neuropathy: a multicenter study. Am J Ophthalmol Case Rep 2021 23 101111. (10.1016/j.ajoc.2021.101111) 34113737 PMC8170359

[72] Tamhankar MA, Pradeep T, Chen Y, et al. Real-world experience with teprotumumab in patients with dysthyroid optic neuropathy. J Neuro Ophthalmol 2024 44 74–79. (10.1097/wno.0000000000001994) PMC10855992 37751310

[73] Carretti AL, Kielwasser G, Borson-Chazot F, et al. Teprotumumab treatment in patients with steroid and surgery-resistant dysthyroid optic neuropathy: a case series. Thyroid 2025 35 1202–1207 Online ahead of print. (10.1177/10507256251372194) 40876911

[74] Habroosh FA, Albrashdi SS, Alsaadi AH, et al. Tocilizumab use for optic nerve compression in thyroid eye disease: a prospective longitudinal cohort. Int Ophthalmol 2024 44 222. (10.1007/s10792-024-03143-4) 38717530

[75] Marcocci C, Bartalena L, Tanda ML, et al. Comparison of the effectiveness and tolerability of intravenous or oral glucocorticoids associated with orbital radiotherapy in the management of severe Graves’ ophthalmopathy: results of a prospective, single-blind, randomized study. J Clin Endocrinol Metab 2001 86 3562–3567. (10.1210/jcem.86.8.7737) 11502779

[76] Menconi F, Marinò M, Pinchera A, et al. Effects of total thyroid ablation versus near-total thyroidectomy alone on mild to moderate Graves’ orbitopathy treated with intravenous glucocorticoids. J Clin Endocrinol Metab 2007 92 1653–1658. (10.1210/jc.2006-1800) 17299076

[77] Moleti M, Violi MA, Montanini D, et al. Radioiodine ablation of postsurgical thyroid remnants after treatment with recombinant human TSH (rhTSH) in patients with moderate-to-severe graves’ orbitopathy (GO): a prospective, randomized, single-blind clinical trial. J Clin Endocrinol Metab 2007 99 1783–1789. (10.1210/jc.2013-3093) 24432992

[78] Cosentino G, Lanzolla G, Comi S, et al. Ablative versus conservative approach for hyperthyroidism treatment in patients with Graves’ orbitopathy: a retrospective cohort study. Thyroid 2025 35 298–306. (10.1089/thy.2024.0633) 39909466

